# Method to Increase Dependability in a Cloud-Fog-Edge Environment

**DOI:** 10.3390/s21144714

**Published:** 2021-07-09

**Authors:** Ovidiu Petru Stan, Szilárd Enyedi, Cosmina Corches, Stelian Flonta, Iulia Stefan, Dan Gota, Liviu Miclea

**Affiliations:** 1 Department of Automation, Faculty of Automation and Computer Science, Technical University of Cluj-Napoca, 400114 Cluj-Napoca, Romania; ovidiu.stan@aut.utcluj.ro (O.P.S.); cosmina.corches@aut.utcluj.ro (C.C.); iulia.stefan@aut.utcluj.ro (I.S.); dan.gota@aut.utcluj.ro (D.G.); liviu.miclea@aut.utcluj.ro (L.M.); 2 Technical College “Iuliu Maniu”, 455300 Simleu Silvaniei, Romania; sflonta@colim.ro

**Keywords:** dependability, secure group communication, vertical resource management, cloud-fog-edge, fog-based access control, decentralized environment, edge computing

## Abstract

Robots can be very different, from humanoids to intelligent self-driving cars or just IoT systems that collect and process local sensors’ information. This paper presents a way to increase dependability for information exchange and processing in systems with Cloud-Fog-Edge architectures. In an ideal interconnected world, the recognized and registered robots must be able to communicate with each other if they are close enough, or through the Fog access points without overloading the Cloud. In essence, the presented work addresses the Edge area and how the devices can communicate in a safe and secure environment using cryptographic methods for structured systems. The presented work emphasizes the importance of security in a system’s dependability and offers a communication mechanism for several robots without overburdening the Cloud. This solution is ideal to be used where various monitoring and control aspects demand extra degrees of safety. The extra private keys employed by this procedure further enhance algorithm complexity, limiting the probability that the method may be broken by brute force or systemic attacks.

## 1. Introduction

The concept of Cloud-Computing has matured a lot in recent years 1. It underlines the fact that all resources, services, and data hosted on the Internet must be available for use whenever more sophisticated services are necessary to be developed and to be provided. Therefore, we can say that omnipresent access, mixed resources, and on demand resource or service delivery in a safe and secure environment are at the base of Cloud-Computing features.

At the same time, the concept of Internet of Things (IoT) has emerged, evolved, and reached reality and maturity. IoT is a concept that defines a world in which all objects (cars, lighting systems, home appliances, and others) are connected to each other via the Internet and through heterogeneous access networks that will generate large amounts of emerging and versatile data as well as many services [1,2,3]. The connected devices may be sensors, actuators, and any other device or object that can be connected, monitored, operated, and controlled. Services will lead to an intelligent, sustainable, and inclusive society and economy. IoT has become one of the most challenging research topics and offers an amazing number of opportunities for business. The media generally focus their attention on the consumer-related Internet segment. There is no doubt that consumer products have an important place in the IoT universe, but they remain, a niche. However, the IoT will have profound implications for all levels of business operations, regardless of the industry type [4,5].

IoT represents the future and the vision of the near future where all the world is connected. At the same time, we must realize that if we want to achieve this, account must be taken of the omnipresent accessibility and connectivity, the management of users and connected devices, the optimal use of resources, and the customization of the services offered based on the users’ preferences and wishes [6]. All these features must be provided in a reliable and scalable environment. Meanwhile, we can also say that IoT is critical to Cloud-Computing and that the convergence of these two paradigms offers huge opportunities for both technologies. Cloud-Computing is based on sharing resources and maximizing their use regardless of location, two key requirements for IoT solutions. Additionally, when talking about IoT solutions, these must be accessible from anywhere and anytime.

In view of the foregoing, we may state that to provide reliable IoT solutions, efficient and scalable resource delivery where and when necessary, the two paradigms need to merge, and Cloud-Computing features are critical to IoT. Therefore, new security issues arise [7,8], but the security research of Cloud-Computing systems is far from being mature [9]. What should be mentioned is that the specific security features in the Cloud are not yet known. Many believe that this domain has few specific requirements and the current security features and existing practices such as encryption, firewalls, digital signatures, can be easily adapted to solve the Cloud-Computing security aspects [8].

However, many industries’ actors have reported that there are various types of threats and that mechanisms, other than conventional ones, need to be developed [10]. In fact, it is unlikely that Cloud-Computing itself will create security problems. These security issues that may arise are not necessarily only technological, they stem from reliability and confidence needs, and from lack of clear information about Cloud security [11].

The production and utilization of on-line resources is a unique characteristic of the Cloud-Computing paradigm. Fog Computing operates on the Edge of the network in order to conserve bandwidth, while the Edge handles data at the verge of the Cloud. Empowering the Edge computation in a Cloud-Fog-Edge environment lowers and reduces the distance data must travel across the network. Therefore, research into this field is necessary due to the unique connection between Cloud, Fog, and Edge computing. This paper’s major contributions are to evaluate and to offer a strategy for increasing dependability in this context. We succeed to clarify how and why the security is an important part of the dependability concept and how one can obtain a vertical management of system resources without overloading the Cloud in a safe and secure environment.

The remainder of this paper is structured as follows. Section 2 presents the related technology, problem finding, and a proposed method to bind security to the dependability concept. Section 3 introduces the proposed method with its algorithms and design. The simulation model and numerical results of the proposed method are discussed in Section 4. Finally, Section 5 concludes the paper.

## 2. Materials and Methods

With the emergence of IoT, Cloud-Computing, humanoid robots, and intelligent robot services, more and more studies have been conducted on robot control and on how robotics have intersected with IoT [12,13]. Two aspects need to be considered for robot control. The first concerns the limitation of resources managed by the robot’s system due to large volume data constraints and costs [13]. The second aspect concerns the cost of integrating robots with IoT.

All IoT devices must be aware, autonomous, and actionable. The awareness refers to the fact that they have all types of sensors and can sense the surrounding environment. The autonomous feature refers to the possibility of automatically transmitting data to other devices, but simultaneously also to the Internet. These features are likewise linked to the fact that someone/something can monitor all the collected information with respect to the awareness feature. The last characteristic, the actionable feature, takes into consideration the fact that these devices must have embedded in them some kind of analysis capability in order to control their sensors/actuators. The control part can be done automatically or can be based on the requirements of the supervisors that monitor the data.

**Figure 1:**
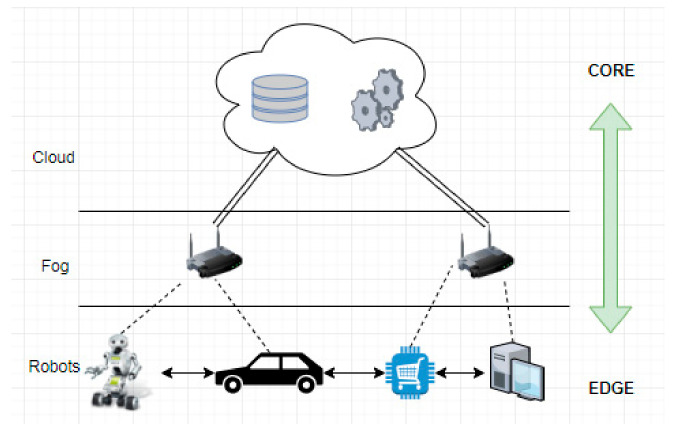
Overall Cloud-Fog-Edge architecture.

Based on all the above-mentioned, we wanted to analyze how Cloud-Computing processes can be brought to the Edge area as much as possible, but at the same time, in a safe and secure environment (Figure 1).

Robots can range from humanoids to sophisticated self-driving automobiles or IoT devices which gather and interpret sensor data locally. Each of the robots must be able to communicate with the others, directly if they are close enough, or through the Fog access points (AP). These robots generate all sorts of data, such as equipped sensors data (light, temperature, gas, etc.), localization information, multimedia data, engine data, and others. In essence, the robots represent the Edge level of our architecture. In the Cloud, all the information and data generated by the robots are collected and stored, but at the same time, the Cloud is also used to process the information.

The proposed Cloud-Fog-Edge architecture takes into consideration the fact that resource awareness must exist vertically [6,14,15]. Through this, the authors desire to obtain the intelligent reduction of the data amounts transmitted from the Edge areas (robots, IoT devices, etc.) through the Cloud [15]. Moreover, this architecture is based on a highly parallelized computing paradigm and needs a decentralization of the analysis algorithms.

The main focuses of this paper are the Fog and Edge areas because, amongst other IoT array of devices, a large set of data is produced, and we want to obtain a middle point between the source of data origin and the top Cloud infrastructure. Through the proposed infrastructure, we should be able to filter, process, and aggregate the data before sending them to the Cloud. With this method, we should be able to offer a highly available compute solution, nevertheless with efficient, reduced resources and in a safe and secure environment.

### 2.1. SEcube™ Open Security Platform

**Figure 2:**
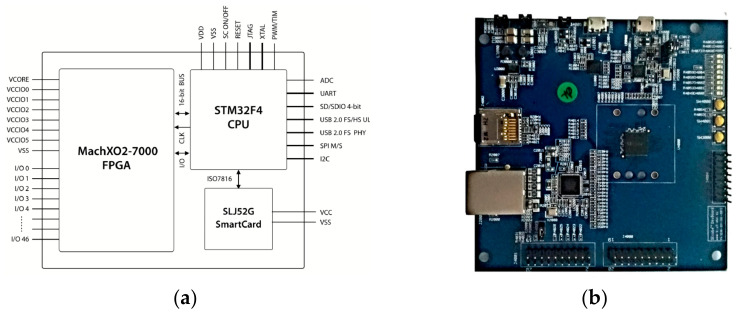
(**a**) SEcube™ Block Diagram. (**b**) SEcube™ Devkit.

The SEcube™ (Secure Environment cube) Open Security Platform (Figure 2) is an open-source security-oriented hardware and software platform, designed and constructed with ease of integration and service-orientation in mind. The hardware part of the platform was designed by Blu5 Group [16], and the software libraries are provided by an international cooperation within European research institutions [17].

The major hardware products are the chip, the development board (devkit), and the USB stick. The SEcube™ chip is the main hardware component, and both the devkit and USB Stick are designed around it. The Development Board provides several communication protocols as well as debugging capabilities. For the final product the board would be of course too inconvenient to carry, and instead the USB Stick is preferred.

The SEcube™ chip integrates three key security elements in a single package: a fast floating-point Cortex-M4 CPU, a high-performance FPGA, and an EAL5+ certified Security Controller (Smart Card). These elements, in conjunction with a set of custom software libraries, allow developers to implement highly reliable security applications [18,19]. The SEcube™ chip can be easily integrated in any project due to the communication protocols available (USB, UART, Ethernet, JTAG).

One of the innovating aspects is the fact that the chip also includes a true random number generator which relies on 240 noise seeds, all physical and therefore unpredictable, allowing the creation of true random noise. Additionally, the user can choose what type of noise they want to generate, for instance, white or Fourier noise.

**Figure 3:**
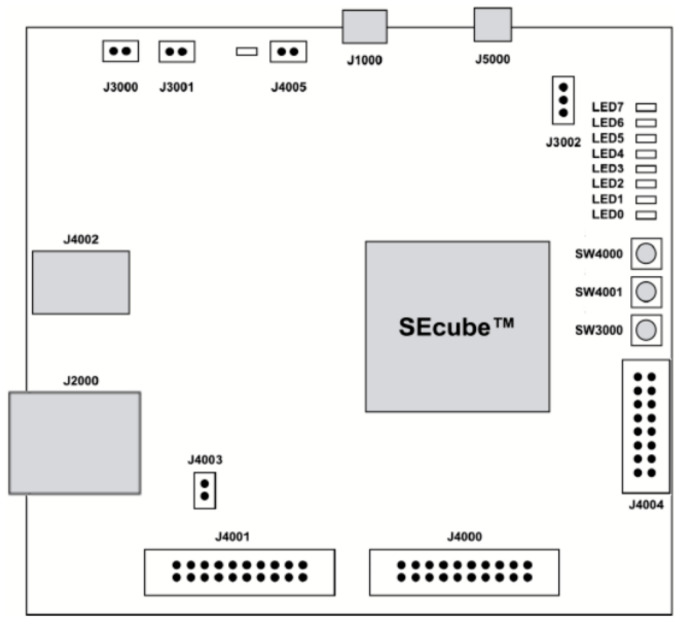
The main peripherals in the SEcube™ devkit.

Figure 3 shows the simplified SEcube™ architecture. The development board integrates the SEcube™ chip with several peripherals that allow the user to easily communicate with, program, and debug the chip. The main peripherals in the SEcube™ devkit are: J1000: USB 2.0 to UART, J2000: Ethernet 10/100 socket, J4000: SEcube™ embedded FPGA and CPU GPIOs, J4001: SEcube™ embedded CPU JTAG, J4002: microSD card, J4004: SEcube™ embedded FPGA and CPU GPIOs, J5000: USB 2.0 High Speed, LEDx: Leds, SWx00y: Switches [20].

From the developer’s point of view, the APIs have been implemented targeting two nested environments depending on where the code physically runs. Thus, there is a Device-Side where the basic functionalities are included and executed on the embedded processor. The Host-Side environment has all the necessary library functions that need to be executed on the host PC. Additionally, this layer provides the interface capable of calling the services and processes residing on the embedded processor.

From architectural point of view, the Host-Side Libraries have been implemented, targeting four hierarchical abstraction levels. The first one (Level 0) is used for Communication Protocol and Provisioning APIs. The second level, (Level 1) holds all the basic Security APIs. The last levels (Level 2 and Level 3) are used for intermediate and, respectively, advanced Security APIs. At every level, each component represents a “service” for the upper level and relies on “services” provided by the next lower level, only.

The Device-Side Libraries only have the lower two levels of abstraction, and each of these levels communicates with its Host-Side counterpart.

### 2.2. Dependability and Security within Cloud System

The Cloud-Computing paradigm can be viewed as a large, distributed computing architecture, whose applications must be accessible from anywhere and anytime. Hence, a Cloud-Computing architecture must provide services complying to a high availability, high fault tolerance, and a dynamic extensibility feature. All the characteristics stated above represent the foundations of the dependability property.

**Figure 4:**
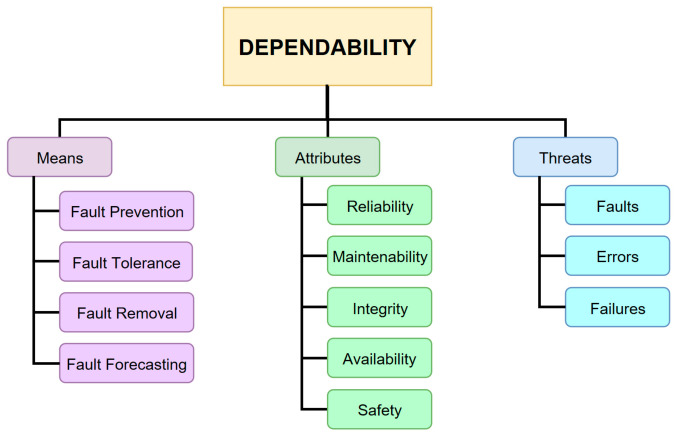
Dependability tree.

The term “dependability” is more and more common in the life cycle of a system. In the literature, there is not only a singular, unique definition of dependability. The Technical Committee of the International Organization for Standardization asserts that dependability is a tool used to measure performance of reliability, maintainability, and maintainability support [21]. Another widespread definition of dependability states that it is the property of a system to prevent it from unexpected or catastrophic damage [22] or the fact that it represents the ability of a system to provide the necessary specific services that can be reliably trusted [23,24]. To have a general view of dependability, we must consider not only the attributes of but also the threats to and the means by which the dependability is attained, as shown in Figure 4.

**Figure 5:**
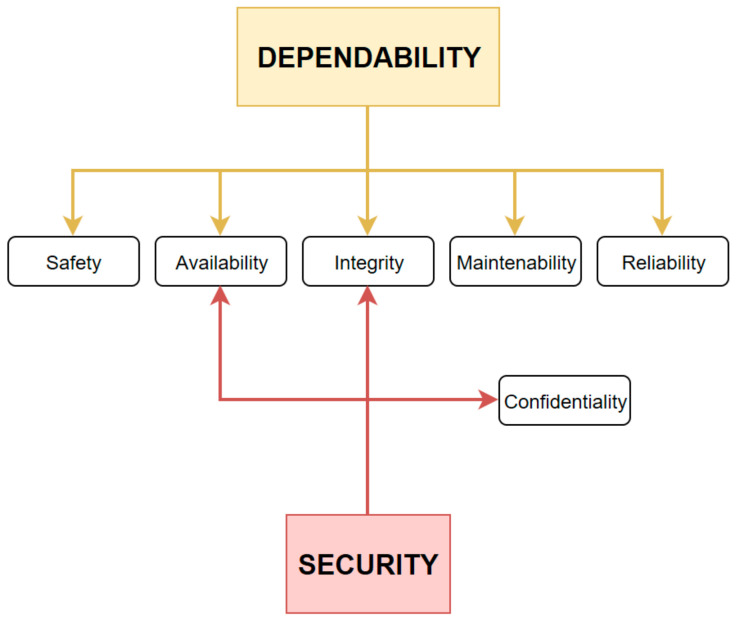
Hook-up dependability and security.

When it comes to analyzing the dependability of a system, we must consider the following six attributes: reliability, availability, integrity, confidentiality, safety, and maintainability [23,24,25]. As one can see, security is not considered being an attribute of dependability. In fact, security is defined by several factors such as preventing data disclosure to unauthorized persons, unauthorized modification or deletion of data, destruction of their integrity. In Figure 5, one can see the link between dependability and security.

To work on dependability of Cloud-Computing systems, we must take into consideration the attributes of recoverability [25], because in this manner, we can quantify the dependability of a system from different perspectives [24].

In the literature, many others have approached the Cloud security topic, such as A4Cloud FP7 Project [26], Cloud Broker Architecture [27], or Phantom [28]. Prokhorenko et al. even though they address the area of data security and trustworthiness, in order to improve the architectural resilience in Cloud, Fog, and Edge systems, all points of identification, authorization, and authentication are made in the Cloud. They do not have a mechanism for extending these resilience mechanisms to the Fog or Edge area of the system [26].

Abderrahim et al. provides a broker architecture with trustworthy qualities committed to Cloud services in which the fault management is included. That broker is an intermediate between the customer and supplier, the obligation to negotiate contract terms and the release of tasks not returned to each of the parties. Therefore, they address the fault tolerance area of dependability [27].

Inside the Phantom project, the authors have succeeded to ensure the adequacy and availability of Cloud subsystems. Phantom uses a fault simulation in the regular operation periods to “disrupt” the Cloud while monitoring and profiling the end user’s availability of service. When Phantom identifies a problem node or an untrusted node, it stops the communication process with it. All data processes are carried out in the Cloud and the resources are thus not vertically handled [28].

As can be seen above, their focus and approach are different from what we propose in this paper and from our aim. Our goal is to provide a safe and secure environment in which the Cloud is not overwhelmed by data and to empower the Fog and Edge with computation.

## 3. Proposed Solution

The chosen solution to validate our concept builds on two previously developed projects: a smart environment monitoring beacon [29] and a remotely operated mobile robot with live camera feed [30]. The multimedia data transmitted by the robot is taken to the Fog by AP and together with the information regarding the PWM and the status of the used current are forwarded to the Cloud where now they are just stored without being processed.

We have chosen to use the information provided by the beacon installed in different rooms and, depending on the room temperature, to change the speed of the robot movement without the user choosing this option from the user interface. The value of the room temperature was sent directly from the beacon to the remotely operated mobile robot using the method presented in Section 3.2.

To demonstrate the proposed method, we tested the provision of access rights at Edge and Fog level, without overloading the Cloud with data. After successfully performing the experiment (the remotely operated mobile robot and the beacon successfully communicate, the speed of the robot was updated according with the room temperature), we wanted to see how fast the proposed method of generating access keys is, with various equipment that can be used in the Fog area. As can be seen in the following experiments, in our tests, we manage to generate, using the true random generation tool provided by SeCube, prime numbers of different sizes (from 6 to 10 digits) and to see how long the process of key generation, encryption, and decryption of messages takes. As we expect and it can be seen in Table 1, Table 2, Table 3 and Table 4, the times increase depending on the size of the randomly chosen prime number, but, still even at a 10-digit number size, the time required to generate the keys is sufficient to provide protection in data communication. These keys change at a pre-set interval, making it impossible or difficult to break it using the brute force-method.

### 3.1. Overall Proposed Architecture

**Figure 6:**
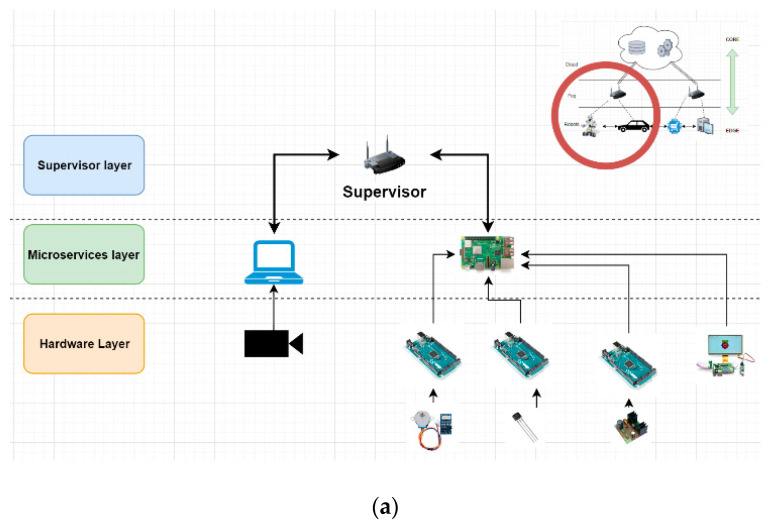

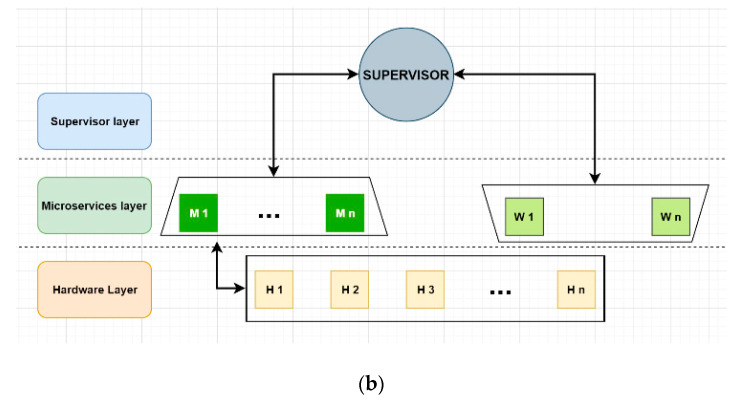
Multilayer FOG architecture: (**a**) binding the camera remote operated robot structure to the proposed Fog-Edge architecture; (**b**) Fog-Edge multilayer abstractization.

As one can observe in Figure 6, we will only address the Fog-Edge area for which we propose an architecture based on three levels. In the lowest level, the hardware level, there are the devices that can produce data (sensors) or can receive commands (stepper motors, motors etc.). The main function of this level is data collection and direct interaction with the environment. The second layer, the microservices layer is the superior layer of the hardware. Here, an entity is a microservice able to communicate with the hardware, the Cloud or the supervisor. The supervisor level has two entities. One is responsible for managing the microservices on this layer and the ones beneath it and the second one is in charge with the connection with the SeCube device and with the process of creating/managing the group key.

The Hardware level devices are named Hardware Components (H) and can be any type of hardware equipment from simple sensors to complex devices, like leap-motion cameras used for stereo vision input. They can have different shapes, dimensions, specific energy consumption requirements, or different communication protocols. These specifications should be integrated in the embedded systems as efficiently as possible, but without neglecting the high scalability needs and with minimum invasive modifications to the drivers and hardware communication software. Usually, most devices offer and API for their libraries which allow the high-level software to easily interact with the hardware in a safe and secure environment.

The entities or the microservices from the microservices level oversee interaction between the hardware and the highest layers/levels. We have divided these microservices into two categories: the ones in the first category interact directly with the hardware component and are named Hardware Monitor (M). These configure and manage the hardware components, collect, and preprocess raw data from the devices, they use low level machine code. Essentially, the monitor is powerfully bound to the I/O because its main attribute is to communicate interoperable with the hardware.

The second type or microservices within the microservices layer offers a high processing power that can do almost everything if the physical resources are available. These are named Workers (W) because they are strictly related to the CPU since they are just processing data. They do not have any connection with the hardware components. The workers process and standardize data from the hardware components and then are responsible for the communication with the Cloud process in order to permanently store the information. Some of the workers could have administrative jobs, like logging or monitoring of resources or even making decisions based on local information/scenario.

The special microservice that oversees the entire system is the Supervisor. It represents the highest level with which the robot can communicate. The Supervisor’s main features are creating, managing, killing, and restarting the microservices.

This multi-level architecture imposes for the components in a level to be able to communicate only with the adjacent levels. The hardware level can only interact with the microservices level, especially with the Hardware Monitors, that are responsible for managing the hardware, collecting data, and translating commands into machine code.

**Figure 7:**
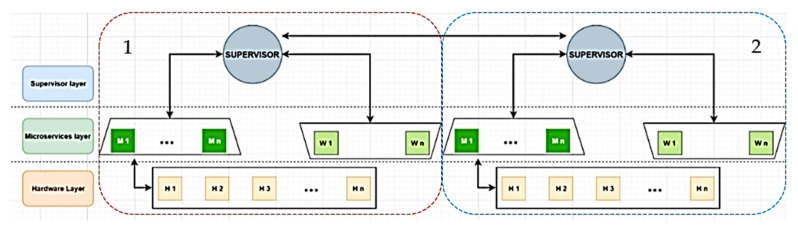
Normal communication process between supervisors.

The microservices cannot directly communicate with each other. They must use the Supervisor in order to do so as shown in Figure 7.

All the above-mentioned functionalities, with respect to the three-layer architecture, were deployed in a real-world experimental setup. Area 1 of Figure 7 represents the remotely operated mobile robot. Its main components are:

- *Supervisor*: Raspberry PI single-board-computer;
- *Microservices Layer*: Arduino Nano ATMEGA328p acting as Hardware Monitor;
- *Hardware Layer*: camera, rotary encoder, motors and L298N Dual H bridge.

Area 2 of Figure 7 represents the smart environment monitoring beacon. Its main components are:

- *Supervisor*: Raspberry PI single-board-computer;
- *Microservices Layer*: Raspberry PI acting also as Hardware Monitor;
- *Hardware Layer*: MCP3008 10-bit Analog-to-Digital Converter (ADC), LM393 vibration pulse sensor, MAX 9814 noise sensor, MQ135 gas sensor, SHT11 temperature and humidity sensor, TSL2591 light sensor, SI1145 UV sensor, and BMP1080 barometric sensor.

The supervisors for each sensor are dictating the operations and steps into measuring or sending data to the FOG. The microservices layer is the one that contains both the measurement trigger operation component as well as the worker component, which executes small operations and data processing. We are introducing a new approach that when a data processing operation is requiring data from other sensors, a supervisor may request data from another one. The supervisor on the right will trigger the measurement operation, the microservices layer will get the data, the workers will process it, and the data are sent back to the left supervisor, which made the data request. The new measurement on the right part can be avoided if the right-side supervisor contains up-to-date information about the sensors it supervises. The left supervisor will provide the necessary data to the workers, and the complete set of results will pe provided by the left supervisor to the Fog. Thus, the number of requests to the Cloud area is reduced in this case by 50%.

### 3.2. Access Rights

Usually, the regular form of many cryptosystems operates between two entities. These entities are the sender and the recipient for the process of encryption. The approach typically uses private and public keys to offer a safe conduit for information privacy and confidentiality. The complexity of most cryptosystems is rooted in the problems of Discrete Logarithm Problem [31,32] or the Integer Factorization Problem [33].

**Figure 8:**
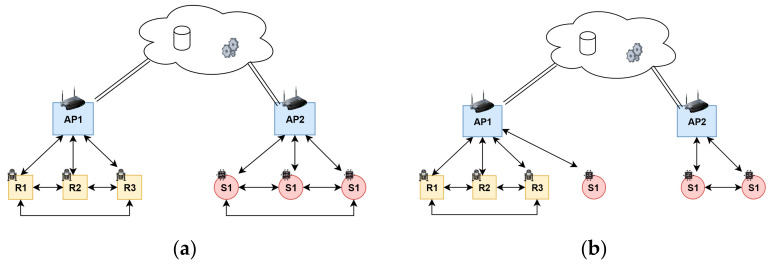
Communication scenario: (**a**) initial position of each robot; (**b**) displacement of a robot from one area of Fog coverage to another area (S1).

A model that allows a device to pass from the connection with the Fog Access Point (AP2) originally assigned to another Fog Access Point (AP1) connection is presented as the following: Figure 8 illustrates layouts, robots access rights, and the event of a robot migrating to a new access point.

To give access to the S1 robot, we modified the classical El Gamal algorithm with a split private key [34]. The description of this algorithm is further presented. Having $\left(Z_{p},\cdot \right)$ as a cyclic group, where the problem of discreet logarithm is difficult to solve [35]. The notation used below is:

(1) $$h^{x}\overset{\mathrm{def}}{=}h^{x}\left(mod\;p\right),$$

**Key generation**: A trust center chosen $g\in \left\{1,2,\ldots ,p-1\right\}$ and $\left\{x_{1},\;x_{2},\;\ldots ,x_{2n+1}\right\}\;\in N^{*}$ distinct, two by two. Following $h_{i}=g^{x_{i}}$, *i* = $\bar{1,2n+1}$ is calculated.

Thus, the public keys are $\left\{p,\;g,\;h_{1},h_{2},\;\ldots ,\;h_{2n+1}\right\}$ and the divided private key is $\left\{x_{1},\;x_{2},\;\ldots ,\;x_{2n+1}\right\}$, submultiples with one element are called private keys.

**Message encryption**: To encrypt message m, it is necessary to determine the number of entities in the group that will decrypt the message together. The number of entities in the group can be an odd number less than or equal to 2*n* + 1 (if the number of entities is even, then an entity receives two private keys). If, by way of example, the chosen number is 3, then $h_{i}$, *i* = $\bar{1,3}$ is used. The value $y\in N^{*}$ is chosen randomly and furthermore we compute $c_{1}=g^{y}$, $c_{21}=mh_{1}^{y}$, $c_{22}=mh_{2}^{y}$, $c_{23}=mh_{3}^{y},\;$and $c_{2=}(c_{21\cdot }c_{23\;})/c_{22}$ resulting in the encrypted message (*c*_1_, *c*_2_).

**Decryption of the message**: If chosen, each entity receives $x_{1}$, $x_{2}$, $x_{3}$, which are their private keys in the key generation phase. Using the encrypted message (*c*_1_, *c*_2_) together, we calculate as follows:

(2) $$\left(c_{2}\cdot c_{1}{}^{x_{2}}\right)/(c_{1}{}^{x_{1}}\cdot c_{1}{}^{x_{3}})=m,$$

and the message is decrypted.

Hereinafter, we describe the protocol to be followed in the context of this algorithm.

The *El Gamal encryption algorithm* with a split private key involves three steps: generating the keys by a trusted center, encrypting the message, and decrypting the encrypted message.

The *Trust Center* can generate a set of keys (public and private) or multiple sets. It can assign all group keys to the private keys or a subset of the private key set. Of course, public keys do not have to be assigned, they are public. Depending on the situation, private keys can be used individually or in groups by entities. If needed, a trust center can change the set of keys.

**Figure 9:**
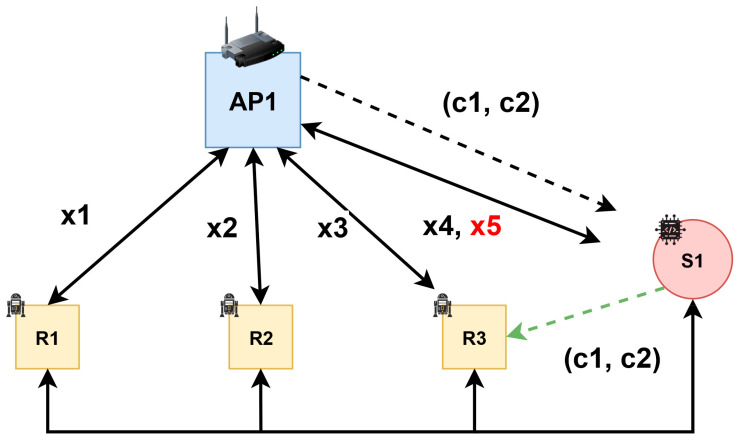
Group membership validation process.

In the concrete situation presented above, AP1 is a trusted center, so it generates the keys of the algorithm. It is also the entity that encrypts the information m. The encrypted form of m is (*c*_1_, *c*_2_), which is issued periodically. When S1 captures the encrypted information (*c*_1_, *c*_2_), it sends this message to the R1, R2, R3 components. They stored the message m in their memory. The components R1, R2, R3, using their private keys *x*_1_, *x*_2_, and *x*_3_, respectively, receive and decrypt the message (*c*_1_, *c*_2_) and then compare the result with the value of m, which they have stored in memory. If the two values coincide, then S1 gets the group access right, as shown in Figure 9. This right is materialized through the process in which the AP1 is transmitting key *x*_4_ or keys *x*_4_, *x*_5_, as appropriate, which it uses as a private key for other information provided by group members. The AP1 can communicate with all members of the group using a private key that he keeps only by himself.

Of course, the number of components initially assigned may be different from three, depending on the situation.

It is important that AP1 periodically emits an encrypted message (*c*_1_, *c*_2_), but choosing another parameter *y* from the algorithm for encryption, so the message (*c*_1_, *c*_2_) will be different every time. The component S1 periodically validating group membership, through the protocol described above, retains access rights. If S1 loses connection with AP1, it will not be able to validate group membership, so it will lose access rights.

## 4. Results

The proposed method was implemented in C and the SEcube™ board was linked to different system configurations, as shown in Table 1. These systems were tested at Fog level in order to check the accuracy and the performance.

### 4.1. Algorithm Accuracy

In the first proposed scenarios, we chose as a prime number *p* = 400,093 and we increased the number of entities in the group starting with three entities until 999 entities, to check the algorithm’s correctness (the decrypted message must be the same with the encrypted message). During encryption and decryption, the test code also documented the time of operations as shown in Table 2.

**Figure 10:**
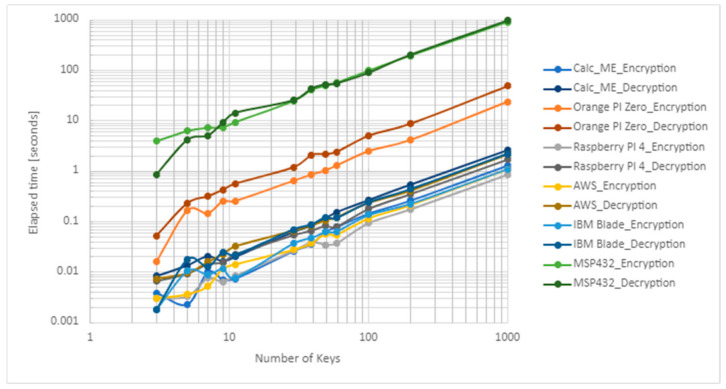
Encryption/decryption elapsed times for *p* = 400,093.

All the experiments shown in Table 2 were successful and the algorithm accuracy did not change regardless the number of entities simulated, and every time the message was successfully encrypted and decrypted. One can see in Figure 10 the fact that the processing time of the algorithm increases with the number of entities in group. The increase is because of the extra factors that both in the encryption and decryption process must be considered.

### 4.2. Algorithm Performance

In order to evaluate the algorithm performance with regard to the value of the prime *p*, an experiment was conducted which injected diverse and growing prime values into a process with a fixed number of senders (3 and respectively 101). Table 3 and Table 4 illustrate the test result, where the prime number value utilized is noted as well as the encryption and decryption time.

**Figure 11:**
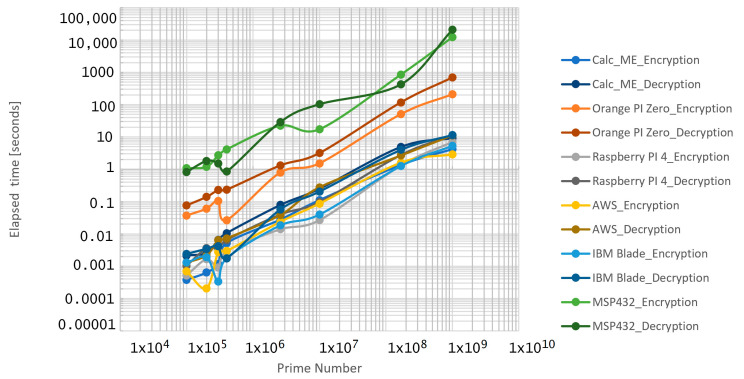
Encryption/decryption elapsed times for three entities in the group.

In the first set of the experiment, where the number of entities within the group is equal with 3, and when the prime value *p* is increased, the results from Table 3 indicated a normal increase in both the encryption and decryption processes. There are other cases in which, despite the considerable disparity in primes, there are no substantial variations in processing times of the algorithm. These are rare occurrences, which may occur when computer processing space is quite minimal in the parameters other than the Prime Modulo. More specifically, the value of the random keys that senders are used as exponents for the generator to generate public keys may be ascribed. Random keys exponentially enhance the processing duration, or reduce the processing period considerably if relatively small as one can see in Figure 11.

**Figure 12:**
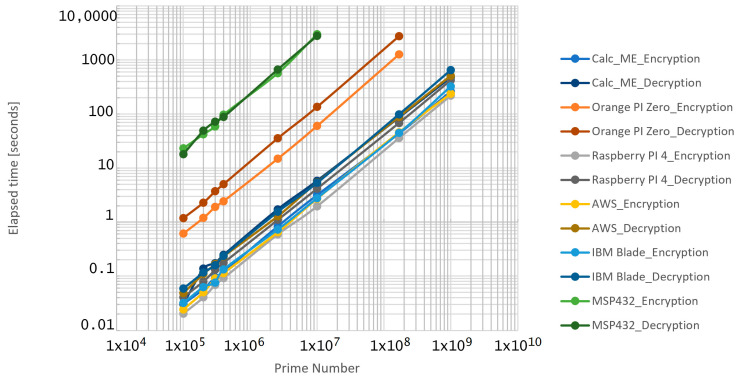
Encryption/decryption elapsed times for 101 entities in the group.

The second set of experiments was designed to test the performance of the algorithm, where the number of entities within the group is equal to 101, and when the prime value *p* is increased, Table 4 and Figure 12 indicated a dramatic increase in both the encrypting and decryption process times. Moreover, as the prime number increased, clearly some of the devices used in the experiments (Orange PI Zero and MSP432) have exceeded their processing capabilities and there were no longer able to encrypt or decrypt the message.

## 5. Discussion

One of the most important issues put forward by this research is the scenario through which resources are vertically partitioned because there are not yet enough algorithms of data analysis. This leads to the problem of preserving the features and accuracy of the centralized parts while using decentralization algorithms to efficiently communicate or process data and resources.

The algorithms used by good password managers are usually standard ones, meaning they are the state-of-the-art, and therefore sturdy. The weak points of the system may be in the master password and in the application being corrupted. A hardware-based manager boosts the security of the system by improving these two points.

The above protocol is not a genuine digital signature because it does not have all the properties of the electronic signature but can be used to connect to/disconnect to/from an Access Point. It also gives the ability to communicate in an encrypted manner with the group members at the same Access Point. Encrypted communication can be done using the El Gamal asymmetric algorithm, El Gamal with the El Gamal split or combining a private key with a symmetric algorithm. The advantage of the protocol is that a simple calculation provides access to the group and at the same time receives a key to communicate in an encrypted manner.

A hardware-based manager uses a two-factor authentication method. In order to encrypt/decrypt the data, two elements are required: a master password and a portable and unique device which is connected to the host machine (the user’s computer, for instance). Therefore, even if an attacker has access to the encrypted data, without the device they cannot even start trying to crack the master password.

Regarding the second point, in plenty of cases the portable device is the one doing all the actual encryption/decryption of data. The host machine is only used to provide the graphical user interface so the user can enter their master password, and to display their protected passwords. As the portable device is custom designed to be as secure as possible, it is much harder to corrupt than an OS or a software application.

Fundamentally, it acts as the password manager’s hardware device, and oversees authenticating the user and encrypting/decrypting the data.

In our opinion, the main advantage derives from the fact that the proposed method’s security is predicated on the performance and efficacy of the discrete logarithm problem [36]. The security issue depends on the size of the first module and the private keys used by the sender and receiver. In the case of the prime, the bigger it is, the more complicated the discrete logarithm problem becomes and therefore the more secure the implementation of our method. Private keys generated for the entities within the group also improve and increase the security of our method. When these keys are kept secret and change periodically, the encrypted text will be very difficult to break, and when there are more private keys, the level of security also increases proportionally.

Another advantage worth mentioning is the capability and versatility of the El Gamal algorithm to employ Elliptic Curve encryption, which ensures smaller/shorter keys with the same level of security.

During our experiments, we found that if the prime number is too big its binary representation might be problematic. This can be considered as a disadvantage and can be observed in Table 4 where we present the encryption decryption times elapsed for generating 101 keys with different values of the chosen prime number *p*. As one can see, when we used big numbers (e.g., *p* = 1,000,000,007), the computing capability of Orange PI Zero and MSP432 was inadequate. However, in our experiments we considered 101 Supervisors, but in a real-world deployment there are considerably fewer.

One notable result is that the single-board computer Raspberry Pi 4 yielded similar or better times than some servers, the IBM blade HS22, for example. The possible explanation we found is that although the latter is a high availability, robust system, it was launched in 2009, and its processors—Intel Xeon E5506—are from the same year. The PI’s processor, Broadcom BCM2711 (Cortex A72), is slower, but is more efficient. Additionally, we used only one thread for all the calculations, thus the dual-Xeon system’s sixteen thread capability did not offer it any advantage. Additionally, the program contains very few conditional branches, therefore the Xeon’s advanced branch prediction system—arguably better than the Cortex A72’s—did not help it gain an advantage.

Obviously, there are optimization options for the GCC compiler, for both platforms, and there also are program code optimizations for best performance, however we used the default settings everywhere, in order to keep the comparisons fair. As an aside, especially converting the program into a multi-threaded one (pthread on GCC), or even using a shell script to run multiple parameterized instances in parallel, for different key calculations, it would run significantly faster on most of the tested platforms. In our opinion, the biggest advantage would be the response time on the inexpensive and slowest four-core platform of the tests, the Orange Pi Zero.

## Figures and Tables

**Table 1:** Systems’ configuration used for testing the method.

	Specifications	RAM	OS	CPU		
Generic Name				Architecture	Model Name	Max MHz
Calc_ME		4.6 GB	Kali GNU/Linux Rolling 2020.1	x86_64	i7-8550U	1991
AWS		1 GB	Amazon Linux 2	x86_64	Xeon E5-2686 v4	2300
Blade HS22		192 GB	Debian Linux 10 x64	x86_64	Xeon E5506	2128
Raspberry PI 4		8 GB	Raspberry Pi OS 64bit beta 2021-05-07	aarch64	Cortex-A72	1500
Orange PI Zero		512 MB	Armbian 21.02.3 Buster	armv7l	Cortex-A7	1008
MSP432		32 kB	N/A	armv7E- M	ARM Cortex-M4F	48

**Table 2:** Encryption/decryption elapsed times for using as a prime number *p* = 400,093.

	Number of Keys	Calc_ME	AWS	Blade HS22	Raspberry PI 4	Orange PI Zero	MSP432
Encryption time (seconds)	3	0.003792	0.002950	0.001757	0.002940	0.015715	3.850827
	5	0.002207	0.003519	0.010239	0.003187	0.164177	6.107344
	7	0.009842	0.005058	0.008657	0.007358	0.141691	7.107184
	9	0.006906	0.011490	0.011201	0.006221	0.250919	7.108379
	11	0.007129	0.013822	0.007431	0.008118	0.250277	9.108432
	29	0.025270	0.027014	0.036156	0.025980	0.633163	24.119451
	39	0.034540	0.035821	0.046292	0.038101	0.838590	40.125051
	49	0.060920	0.056324	0.060243	0.034095	1.018220	48.132469
	59	0.075528	0.053660	0.062004	0.036033	1.266727	55.137456
	101	0.141051	0.114034	0.132272	0.091636	2.465663	97.160261
	201	0.254484	0.208343	0.219440	0.170360	4.085383	192.057957
	999	1.255248	1.061463	1.067896	0.829035	23.410290	891.565563
Decryption time (seconds)	3	0.008109	0.007238	0.001757	0.006446	0.051843	0.848784
	5	0.013275	0.009237	0.017464	0.009023	0.227183	4.118981
	7	0.020129	0.015434	0.012378	0.013925	0.310155	4.949387
	9	0.015878	0.022096	0.024552	0.015025	0.421439	9.024027
	11	0.019475	0.031550	0.021267	0.021742	0.553362	14.033328
	29	0.060970	0.064622	0.068585	0.052398	1.171324	25.037525
	39	0.080712	0.081997	0.085996	0.064432	2.056169	43.047829
	49	0.113780	0.103776	0.117924	0.080060	2.140582	51.052091
	59	0.150036	0.118909	0.115510	0.076934	2.352839	54.052200
	101	0.263033	0.231263	0.236785	0.176113	4.985249	88.074300
	201	0.529267	0.400894	0.432287	0.342664	8.543328	200.108416
	999	2.541559	2.105250	2.169411	1.648172	47.411385	950.524400

**Table 3:** Encryption/decryption times elapsed for generating three keys with different values of the chosen prime number *p*.

	Prime Number	Calc_ME	AWS	Blade HS22	Raspberry PI 4	Orange PI Zero	MSP432
Encryption time (seconds)	100,003	0.000379	0.000696	0.001292	0.000522	0.036894	1.085995
	200,003	0.000636	0.000204	0.001898	0.001654	0.06026	1.196319
	300,007	0.001187	0.002765	0.000334	0.000906	0.103421	2.717733
	400,093	0.005112	0.00295	0.001757	0.00294	0.026618	4.135892
	2,580,647	0.027725	0.023476	0.018371	0.01399	0.794401	22.390173
	10,000,019	0.110669	0.086406	0.039759	0.026738	1.519801	17.363993
	168,101,891	1.349116	1.584795	1.266459	1.317992	50.734525	852.846315
	1,000,000,007	4.161788	2.862598	5.33795	7.058216	208.274955	12192.42917
Decryption time (seconds)	100,003	0.002148	0.001267	0.002387	0.001033	0.076179	0.823765
	200,003	0.002548	0.002149	0.003434	0.003622	0.138875	1.808158
	300,007	0.0065	0.006355	0.004163	0.003488	0.227826	1.517216
	400,093	0.010519	0.007238	0.001757	0.006446	0.233285	0.850389
	2,580,647	0.078478	0.038323	0.057052	0.039814	1.313003	29.039554
	10,000,019	0.249443	0.275652	0.203809	0.096067	3.167932	103.077165
	168,101,891	4.94765	2.669504	3.99942	2.76056	117.272115	425.265763
	1,000,000,007	9.293838	11.213604	11.526725	11.157354	689.501209	20920.1880

**Table 4:** Encryption/decryption times elapsed for generating 101 keys with different values of the chosen prime number *p*.

	Prime Number	Calc_ME	AWS	Blade HS22	Raspberry PI 4	Orange PI Zero	MSP432
Encryption time (seconds)	100,003	0.030563	0.024321	0.031882	0.020362	0.612009	23.112728
	200,003	0.057708	0.050566	0.062348	0.040512	1.193771	42.127589
	300,007	0.095519	0.0907	0.075618	0.069198	1.910551	59.135877
	400,093	0.12032	0.114034	0.132272	0.091636	2.437267	97.149856
	2,580,647	0.831227	0.649648	0.725774	0.591443	14.902459	572.404987
	10,000,019	3.144726	2.713392	2.766433	1.96131	60.151246	3019.750651
	168,101,891	43.450642	45.825378	44.493693	35.974791	1272.355727	N/A
	1,000,000,007	261.076881	239.84121	327.192183	220.291081	N/A	N/A
Decryption time (seconds)	100,003	0.030563	0.048631	0.059284	0.040707	1.183834	18.035483
	200,003	0.137199	0.109286	0.116095	0.079779	2.290632	49.049392
	300,007	0.173395	0.166254	0.159118	0.130729	3.735541	72.065493
	400,093	0.246389	0.231263	0.236785	0.176113	5.036589	88.068507
	2,580,647	1.721139	1.281048	1.553726	1.095922	35.489642	663.368155
	10,000,019	5.820214	5.394063	5.345031	4.151329	135.340537	2778.43138
	168,101,891	86.110151	88.65764	98.976979	68.181818	2752.042902	N/A
	1,000,000,007	475.85463	511.402519	653.436455	423.499305	N/A	N/A

## Data Availability

Not applicable.
